# Elevated plasma glypicans are associated with organ failure in patients with infection

**DOI:** 10.1186/s40635-018-0216-z

**Published:** 2019-01-07

**Authors:** Jane Fisher, Adam Linder, Peter Bentzer

**Affiliations:** 1 Faculty of Medicine, Department of Clinical Sciences Lund, Division of Infection Medicine, Lund University, Lund, Sweden; 2 Department of Anesthesia and Intensive Care, Helsingborg Hospital, Helsingborg, Sweden; 3 Faculty of Medicine, Department of Clinical Sciences Lund, Division of Anesthesia and Intensive care, Lund University, Lund, Sweden

**Keywords:** Glycocalyx, Glypicans, Sepsis, Organ failure, Infection

## Abstract

**Background:**

Increased vascular permeability is a key feature in the pathophysiology of sepsis and the development of organ failure. Shedding of the endothelial glycocalyx is increasingly being recognized as an important pathophysiological mechanism but at present it is unclear if glypicans contribute to this response. We hypothesized that plasma levels of glypicans (GPC) are elevated in patients with sepsis.

**Methods:**

Plasma GPC 1–6 levels were measured by ELISA in 10 patients with sepsis and 10 healthy controls as an initial screening. Plasma GPC 1, 3, and 4 were further measured in a cohort of 184 patients with a clinically confirmed infection. Patients were divided into groups of those who had sepsis and those who had an infection without organ failure. To determine whether plasma glypicans could predict the development of organ failure, patients were further subdivided to those who had organ failure at enrolment and those who developed it after enrollment. The association of plasma GPC 1, 3, and 4 with organ failure and with various markers of inflammation, disease severity, and glycocalyx shedding was investigated.

**Results:**

In the pilot study, only GPC 1, 3, and 4 were detectable in the plasma of sepsis patients. In the larger cohort, GPC 1, 3, and 4 levels were significantly higher (*p* < 0.001) in patients with sepsis than in those with infection without organ failure. GPC 1, 3, and 4 were significantly positively correlated with plasma levels of the disease severity markers C-reactive protein, lactate, procalcitonin, and heparin binding protein, and with the marker of glycocalyx degradation syndecan 1. They were significantly negatively correlated with plasma levels of the glycocalyx-protective factors apolipoprotein M and sphingosine-1-phosphate.

**Conclusions:**

We show that GPC 1, 3, and 4 are elevated in plasma of patients with sepsis and correlate with markers of disease severity, systemic inflammation, and glycocalyx damage.

**Electronic supplementary material:**

The online version of this article (10.1186/s40635-018-0216-z) contains supplementary material, which is available to authorized users.

## Background

Sepsis is an increasingly important health problem that continues to have high mortality and morbidity [1]. The breakdown of endothelial barrier function, leading to vascular leak, edema, and organ failure, is central to the pathophysiology of sepsis [2, 3]. The endothelium is covered by a complex extracellular gel of both membrane-bound and more loosely attached proteins and glycans which collectively are referred to as the glycocalyx [4]. The glycocalyx has a number of important functions for vascular homeostasis. It acts as a sensor of shear stress, it provides receptor sites for a number of signaling molecules, it maintains an antithrombotic surface, and by creating a negatively charged fiber matrix, it contributes to the barrier function of the endothelium [5–7].

Proteins with glycosaminoglycan side chains, known as proteoglycans, make up the foundation of the glycocalyx [5]. Most endothelial proteoglycans are secreted, while syndecans and glypicans remain attached to the cell membrane [8]. The four syndecans contain the glycosaminoglycan species heparan sulfate and sometimes chondroitin sulfate and are attached to the cell surface by a transmembrane domain. The six glypicans primarily contain heparan sulfate and are attached to the phospholipids of the membrane via a glycosylphosphatidylinositol anchor [8].

Proteoglycans can be removed from the cell membrane in a process known as shedding [9]. The shedding can be induced by pro-inflammatory factors from the host [10] and from infecting pathogens [11]. Some bacteria also carry enzymes that can directly shed glycocalyx components [12, 13]. Supporting a role for shedding of the glycocalyx in the pathophysiology of the critically ill are observational studies demonstrating plasma concentrations of syndecans are generally higher in non-survivors than in survivors [14–16] and experimental studies showing increased permeability for macromolecules following degradation of the glycocalyx [17, 18].

Whether glypicans are shed in conjunction with syndecans in sepsis is largely unknown at present. To our knowledge only one study has measured glypican levels in severe infections and found that glypican 3 concentrations were higher in patients with acute respiratory distress syndrome (ARDS) than in those with severe pneumonia [19]. The primary aim of this study was to determine whether plasma levels of any of the six glypicans are associated with the presence and development of organ failure in patients with infection. Some of the data presented in this manuscript has previously been published as a poster abstract [20].

## Methods

### Patient enrollment and sample collection—pilot study

The aim of the pilot study was to determine which, if any of the six glypicans, are detectable and elevated in the plasma of patients with sepsis. Plasma samples were collected from ten patients who were admitted to the Clinic for Infectious Diseases at Lund University Hospital (Lund, Sweden). Patients had confirmed sepsis as classified based on presence of systemic inflammatory response syndrome (SIRS) criteria, the presence or absence of organ failure, and the final diagnosis, according to the criteria proposed by the American College of Chest Physicians/Society of Critical Care Medicine [21]. Informed consent was obtained as approved by the ethics committee of Lund University Hospital (Diarie number 2014/741). Plasma from ten healthy controls was obtained with informed consent as approved by the ethics committee of Lund University Hospital (Diarie number: 2013/728).

### Patient enrollment and sample collection—main cohort

**Fig. 1:**
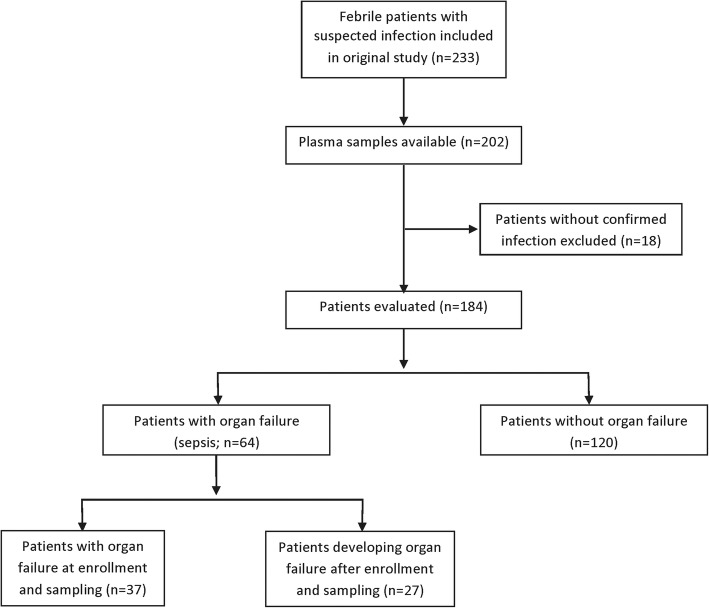
Flow chart describing the study design in the main cohort. The original study is described in [22]

The study design of the main cohort is summarized in a flow chart in Fig. 1. The main cohort was taken from a prospective nonconsecutive convenience sample study of febrile adult patients with clinically suspected infection who were enrolled into the study upon admission to the Clinic for Infectious Diseases at Lund University Hospital (Lund, Sweden) between March 2006 to April 2008 [22]. Plasma samples were collected upon enrollment as described previously, using sodium citrate as the anticoagulant [22]. The protocol was approved by the ethics committee of Lund University Hospital (Diarie number 790/2005), and informed consent was obtained from all patients or their close relatives. Plasma samples were available from 184 patients with confirmed infection. This group of patients is hereafter referred to as the main cohort. Patients were classified based on presence of systemic inflammatory response syndrome (SIRS) criteria, the presence or absence of organ failure, and the final diagnosis, according to the criteria proposed by the American College of Chest Physicians/Society of Critical Care Medicine [21]. Because the Sepsis-3 criteria emphasize organ dysfunction in sepsis [1], we separated the patients into two groups: (1) infection with organ failure (sepsis; *n* = 64) and (2) infection without organ failure (*n* = 120). In some analyses, we further subdivided group 1 into two groups: (a) those that already had organ failure at the time of enrollment and sample collection (*n* = 37) and (b) those that developed organ failure after enrollment and sample collection (*n* = 27).

### Glypican analysis

Plasma concentrations of glypican (GPC) 1–4 were determined using ELISA kits from Cloud Clone Corp. GPC 5 and 6 were analyzed using ELISA kits from Cusabio Biotech. ELISA steps were performed according to the manufacturer’s directions. The standard curve range for the GPC 3 ELISA was extended below the manufacturer’s recommendation to 0.0375 ng/mL after validating that the linearity of the curve was preserved at this concentration (not shown). The limit of detection (LOD) of each assay, as reported by the manufacturer, was 0.32 ng/mL for GPC 1, 0.057 ng/mL for GPC 3, and 0.0142 ng/mL for GPC 4.

The majority of the values were above the LOD of each assay prior to multiplication by the dilution factor. For GPC 1, 31 (16.8%) of data points were below the LOD; for GPC 3, no data points (0%) were below the LOD; and for GPC 4, 12 (6.5%) of data points were below the LOD. Values below the LOD were not modified in any subsequent analysis.

### Measurement of other plasma markers

Plasma concentrations of syndecan (SDC) 1 were determined using an ELISA kit from Diaclone according to the manufacturer’s directions. Heparin binding protein (HBP), interleukin (IL)-6, procalcitonin (PCT), C-reactive protein (CRP) and lactate [22], sphingosine-1-phosphate (S1P) [23], and apolipoprotein M (ApoM) [24] were measured previously in these patients. Briefly, HBP was measured using an in-house ELISA assay [25], IL-6 was measured by a commercial ELISA (Quantikine; R&D Systems), procalcitonin levels were measured with an enzyme-linked fluorescent immunoassay (Biomérieux), and C-reactive protein and lactate analyses were performed on a Roche Hitachi Modular-P [22]. S1P was measured by liquid chromatography coupled to mass spectrometry [23]. ApoM was measured using an in house ELISA [24].

### Statistical analysis

Analysis by the D’Agostino and Pearson K2 omnibus normality test found that plasma GPC 1, 3, and 4 levels are not normally distributed (*p* < 0.01). Therefore, non-parametric analyses were used in all cases. Concentrations of plasma GPC in sepsis and infection without organ dysfunction groups were compared by Mann-Whitney test. Baseline variables were compared by Mann-Whitney test or chi-squared test as appropriate. Receiver operating characteristic (ROC) curves were generated to compare patients who developed organ dysfunction to those who did not.

To adjust for confounding variables, multivariate logistic regression was performed as follows. Potential confounding variables were first compared between groups by a univariate analysis, described in Table 1. Those with *p* values below the chosen threshold of 0.25 were included in the initial model. To reduce the number of variables in the model, a backward elimination approach was used. Variables were removed from the model one by one and those that did not change the parameter estimate of the glypican in question by more than 15% were removed from the model, leaving only the greatest confounding variables. Then, variables that had initially been excluded from the model were then added one by one and included only if they changed the parameter estimate of the glypican in question by more than 15%. Glycocalyx-related factors ApoM, S1P and SDC1 were added to the final model to determine whether they also have any confounding effects.

**Table 1:** Patient characteristics at time of blood sampling (baseline)

	Organ failure (*n* = 64, 35%)	No organ failure (*n* = 120, 65%)	*p* value*
Age, median (IQR)	65 (54–76)	53 (36–71)	0.005
Male, *n* (%)	38 (59%)	55 (46%)	0.080
Site of infection, *n* (%)			
Respiratory tract	15 (23%)	63 (53%)	< 0.001
Urinary tract	19 (29%)	22 (18%)	0.078
Skin/soft tissue	14 (22%)	16 (13%)	0.135
Other	16 (26%)	19 (16%)	0.131
Infection agent, *n* (%)			
Gram positive bacteria	23 (36%)	13 (11%)	< 0.001
Gram negative bacteria	19 (30%)	22 (18%)	0.078
Other bacteria	3 (5%)	12 (10%)	0.210
Virus	1 (2%)	37 (31%)	< 0.001
Unknown agent	18 (28%)	36 (30%)	0.790
Laboratory variables at baseline, median (IQR)			
WBC (×10^9^ cells/L)	12 ± (6–19)	11 (8–14)	0.490
Temperature (°C)	38.9 (38.0–39.6)	38.5 (38–39)	0.018
Pulse (beats per minute)	105 (90–120)	90 (80–102)	< 0.001
Lactate (mM/L)	1.6 (1.2–2.4)	1.0 (0.8–1.4)	< 0.001
CRP (mg/L)	164 (107–235)	77 (26–182)	< 0.001
IL-6 (pg/mL)	493 (106–3300)	0 (0–73)	< 0.001
PCT (μg/L)	5.8 (1.5–15.6)	0.12 (0.05–0.5)	< 0.001

*Groups were compared by chi-square test (categorical variables) or by Mann Whitney *U* test (continuous variables)

Where indicated, *p*-values were multiplicity adjusted using the indicated method. *P*-values below 0.05 were considered significant. Data were analyzed using R (version 3.5.1) for multivariate logistic regression, and using Graphpad Prism (version 7) for all other analyses.

## Results

### Pilot measurement of GPC 1–6

In a pilot experiment, plasma concentrations of GPC 1–6 were measured in the plasma of 10 patients with sepsis and 10 healthy controls. Although GPC 5 expression is largely brain-specific and GPC 2 expression is largely absent from adults [26], sepsis can compromise blood brain barrier function [27] and can sometimes induce expression of fetal isoforms of proteins [28]. Therefore, we considered it prudent to first measure the plasma concentration of all six glypicans. Plasma concentrations of GPC 1, 3, and 4 were higher in sepsis patients than in healthy controls (Additional file 1: Figure S1) while GPC 2, 5, and 6 were below the detection limit of the assays in all patients (not shown). Based on these results, GPC 1, 3, and 4 were chosen for further analysis in the main cohort of 184 patients with sepsis (*n* = 64) or with infection without organ dysfunction (*n* = 120).

### Baseline characteristics of the Lund cohort

Baseline characteristics of the cohort were compared between the groups and are presented in Table 1. Groups differed significantly in their median age, site of infection, infectious agent, and various laboratory variables.

### Plasma concentrations of GPC 1, 3, and 4 in the Lund cohort

**Fig. 2:**
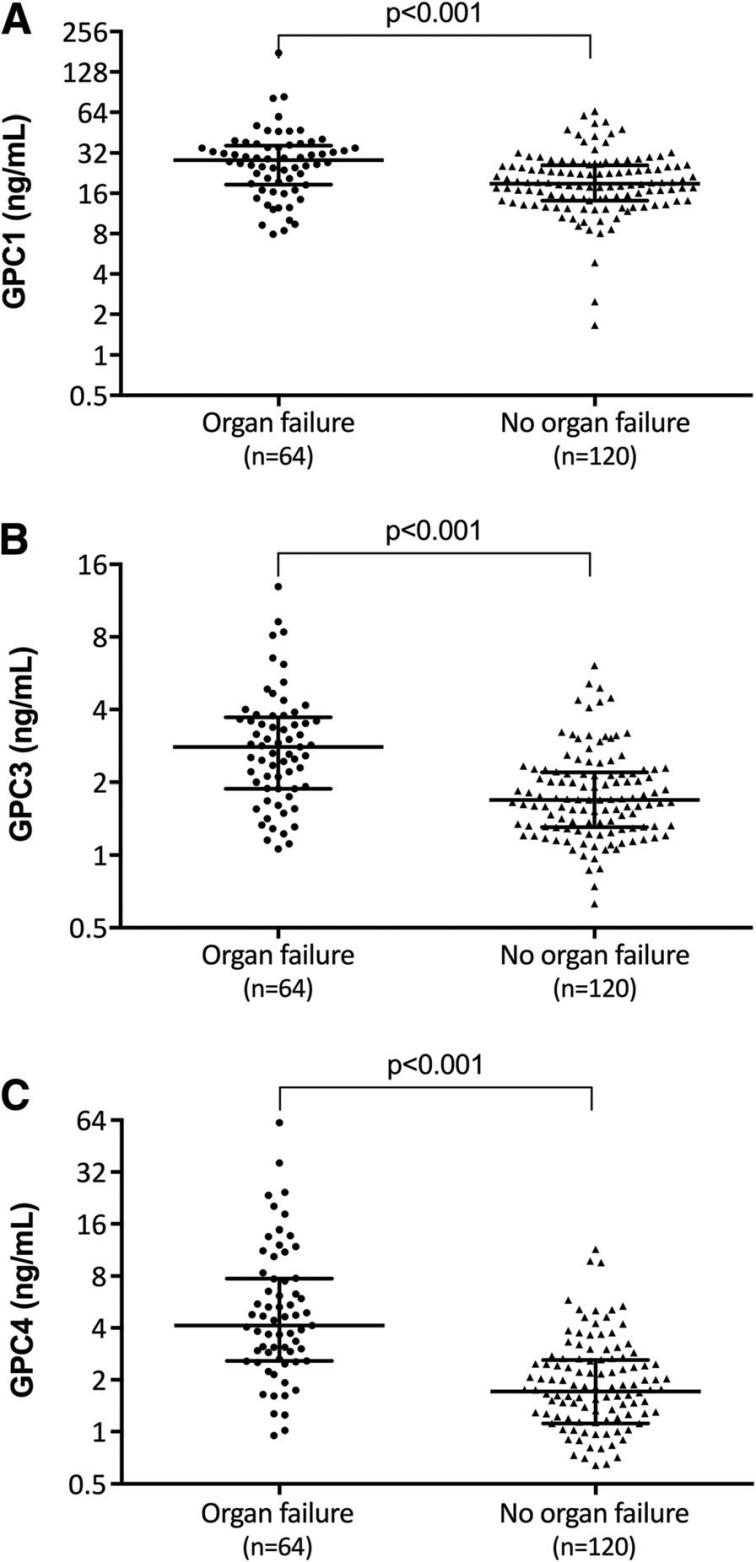
Elevated plasma GPC1, 3, and 4 levels are associated with sepsis. **a** GPC 1, **b** GPC 3, and **c** GPC 4 levels were measured by ELISA in 184 patients with infection who were divided into groups based on the presence or absence of any organ failure. Differences between these groups were determined by Mann-Whitney test

GPC 1, 3, and 4 levels were measured by ELISA in the plasma of the 184 patients in the Lund cohort. Patients with sepsis had higher plasma levels of GPC 1 (28 [18–36] ng/mL vs 19 [14–26] ng/mL; *p* < 0.001), GPC 3 (2.8 [1.9–3.7] ng/mL vs 1.7 [1.3–2.3] ng/mL; *p* < 0.001), and GPC 4 (4.3 [2.6–7.8] ng/mL vs 1.7 [1.1–2.6] ng/mL; *p* < 0.001) compared to patients with infection without organ failure (Fig. 2). In order to adjust for potential cofounding variables, a logistic regression model was built for each glypican using a backward elimination method to reduce the number of variables, using the presence of organ failure as the dependent variable. Variables with no confounding effect were removed and the final models are summarized in Table 2. GPC 1 (odds ratio [OR] = 1.07 [1.03–1.10]; *p* < 0.001), GPC 3 (OR = 1.79 [1.22–2.73]; *p* = 0.004), and GPC 4 (OR = 1.45 [1.17–1.86]; *p* = 0.002) all remained significantly associated with sepsis even when adjusted for these confounding variables.

**Table 2:** Multivariate logistic regression analysis of the association of plasma GPC 1, 3, and 4 with the presence of organ failure and progression to organ failure

Dependent variable	Independent variables in final model	Variable of interest	Coeff.	Odds ratio	95% confidence intervals	*p* value
Any organ failure	GPC1 + WBC + CRP + IL-6 + Gram positive	GPC 1	0.0631	1.065	1.032–1.103	< 0.001
	GPC3 + IL.6 + PCT + Virus	GPC 3	0.584	1.790	1.215–2.727	0.004
	GPC4 + temp + Lactate + PCT	GPC 4	0.368	1.445	1.171–1.858	0.002
Development of organ failure	GPC1 + temp + CRP + IL.6 + Gram Positive	GPC 1	0.0678	1.070	1.021–1.129	0.008
	GPC3 + PCT + Gram Positive + Virus	GPC 3	0.645	1.906	1.219–3.089	0.005
	GPC4 + temp + Lactate	GPC 4	0.332	1.393	1.147–1.842	0.006

*Coeff.* Coefficient (parameter estimate)

### Association of plasma GPC 1, 3, and 4 with the development of organ failure

**Fig. 3:**
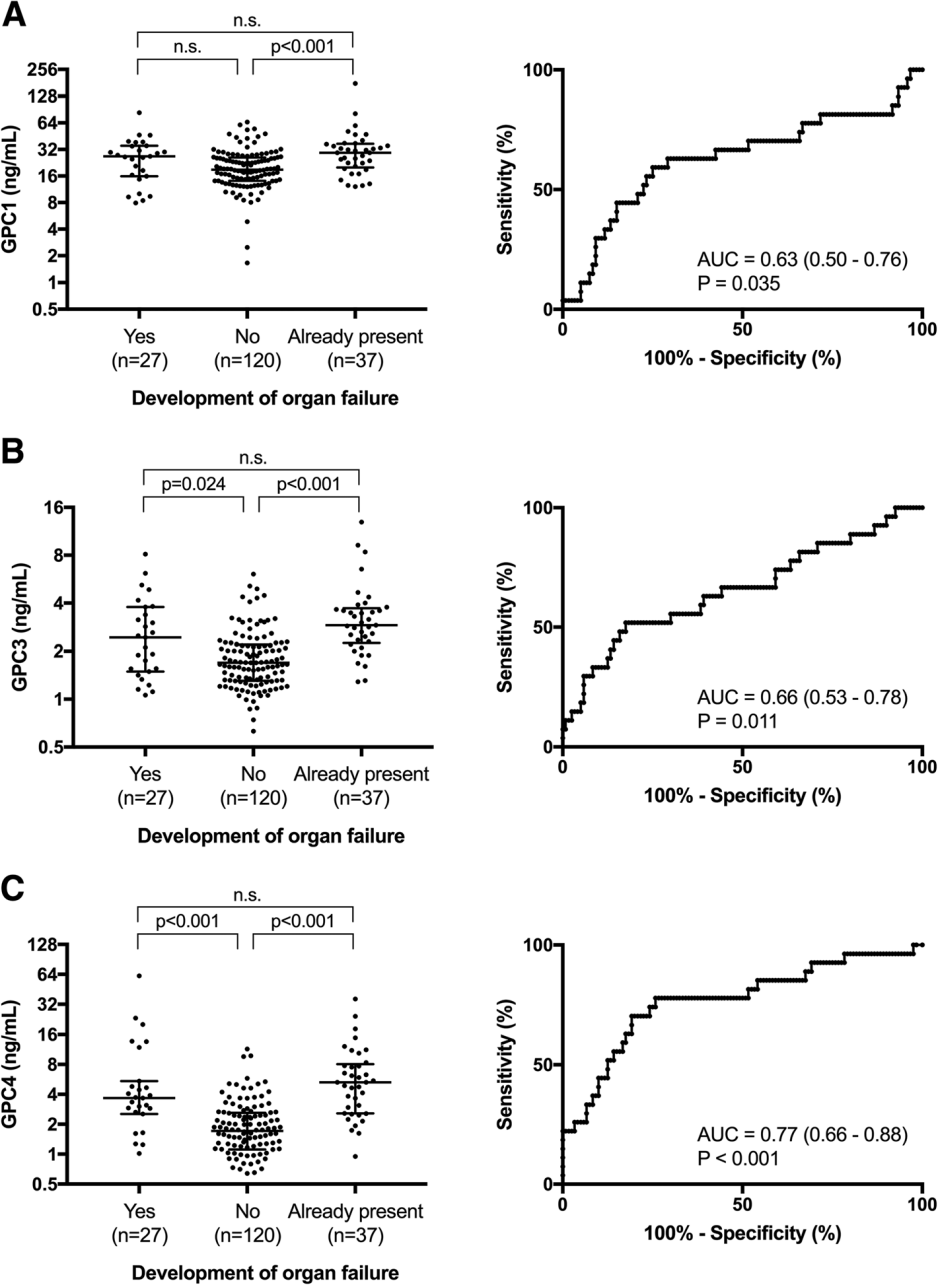
Plasma glypicans are predictive of the development of organ dysfunction. Patients were divided into groups of those who developed organ failure after enrollment, those who never developed organ failure, and those who already had organ failure on enrollment. Plasma levels of **a** GPC 1, **b** GPC 3, and **c** GPC 4 were compared between groups using a Kruskal-Wallis test followed by Dunn’s multiple comparisons test. ROC curves were generated to examine the ability of each glypican to predict the development of organ failure in patients who did not already have organ failure upon enrollment. Area under the curve (AUC) and 95% confidence intervals are indicated on each graph

To determine whether elevated plasma glypican levels could predict the development of organ failure, patients in the sepsis group were further subdivided into two groups: (a) patients who already had organ failure at the time of enrollment and plasma sampling and (b) patients who developed organ failure after enrollment and plasma sampling, and these groups were compared to patients with infection without organ failure. Plasma GPC 3 and 4, but not GPC 1, were significantly elevated in patients who developed organ failure after enrollment compared to those who never developed organ failure (Fig. 3). Receiver operating characteristic (ROC) curves indicated that plasma GPC 1 (area under curve [AUC] = 0.63 [0.50–0.76]; *p* = 0.035), GPC 3 (AUC = 0.66 [0.53–0.78]; *p* = 0.011), and GPC 4 (AUC = 0.77 [0.66–0.68]; *p* < 0.001) had a moderate predictive value for the development of organ failure in patients who did not already have organ failure at enrollment (Fig. 3).

To adjust for potential confounding variables, a logistic regression model was built for each glypican as described before, with the development of organ failure as the dependent variable, excluding patients who already had organ failure at enrollment (Table 2). GPC 1 (odds ratio [OR] = 1.07 [1.02–1.13]; *p* = 0.008), GPC 3 (OR = 1.91 [1.22–2.09]; *p* = 0.005), and GPC 4 (OR = 1.39 [1.14–1.84]; *p* = 0.006) all were significantly associated with the development of organ failure when adjusted for confounding variables.

### Association of GPC 1, 3, and 4 with makers of inflammation and disease severity

Together these results indicate that a severe state of inflammation is associated with higher plasma levels of GPC 1, 3, and 4. Therefore, we examined whether plasma GPC 1, 3, and 4 levels are associated with other markers of inflammation and disease severity across the whole cohort (Table 3). Plasma GPC 1, 3, and 4 were all significantly positively correlated with plasma levels of lactate and PCT. Only GPC 3 and 4 were significantly correlated with CRP and IL-6. Plasma heparin binding protein (HBP), a predictive marker of the development and severity of sepsis [29], was also significantly correlated with plasma levels of GPC 1, 3 and 4.

**Table 3:** Correlation of plasma GPC 1, 3 and 4 with plasma levels of markers of inflammation and disease severity, and with proteins associated with the glycocalyx

	GPC 1		GPC 3		GPC 4	
	Spearman *R*	*p* value*	Spearman *R*	*p* value*	Spearman *R*	*p* value*
Disease severity markers						
HBP	0.20	0.019	0.41	0.001	0.49	< 0.001
WBC	− 0.09	0.38	0.09	0.38	0.13	0.07
CRP	− 0.07	0.38	0.30	0.001	0.44	< 0.001
IL-6	0.18	0.056	0.37	0.001	0.47	< 0.001
Lactate	0.24	0.005	0.33	0.001	0.42	< 0.001
PCT	0.22	0.015	0.40	0.001	0.64	< 0.001
Glycocalyx components						
SDC 1	0.16	0.061	0.35	0.001	0.38	< 0.001
GPC 1						
GPC 3	0.46	0.002				
GPC 4	0.25	0.002	0.56	0.001		
Glycocalyx protective proteins						
S1P	− 0.19	0.037	− 0.04	0.57	− 0.27	< 0.001
ApoM	− 0.10	0.056	− 0.39	0.001	− 0.54	< 0.001

*p values were determined by non-parametric Spearman correlation and adjusted for multiple comparisons by the Holm-Sidak method

### GPC 1, 3, and 4 and glycocalyx shedding

Because inflammatory conditions, including sepsis, are associated with massive shedding of the glycocalyx from endothelial and other cell surfaces, we examined whether GPC 1, 3, and 4 shedding may occur in conjunction with the shedding of other glycocalyx components (Table 2). Indeed, plasma levels of SDC 1 were strongly positively correlated with GPC 3 and 4, but not GPC 1. Additionally, plasma GPC 1, 3, and 4 were strongly correlated with each other, suggesting that they may be shed together as part of a mass shedding of the glycocalyx. The signaling molecule sphingosine-1-phosphate (S1P) and its main carrier protein apolipoprotein M (ApoM) are known to exert protective and regenerative effects on the glycocalyx [30]. Plasma levels of S1P were significantly negatively correlated with GPC 1 and 4, and plasma levels of ApoM were significantly negatively correlated with GPC 3 and 4, suggesting that a decrease of plasma levels of protective S1P/ApoM can be associated with GPC shedding. When these glycocalyx-associated variables were added to the logistic regression models generated previously, they did not greatly change the models (not shown) suggesting that they do not have a confounding effect on the association of plasma glypicans with organ failure.

## Discussion

In this study, we examined whether glypicans are shed during sepsis and whether they are associated with the development of organ failure. So far, studies of glycocalyx shedding in sepsis and inflammatory conditions have focused largely on syndecans, hyaluronan, and heparan sulfate [10]. The four syndecans have been systematically measured in the plasma of intensive care unit patients and showed that only syndecan-1 and 3 are elevated compared to healthy controls [14]. To our knowledge, a similar systematic study of glypican levels in plasma has never been carried out. In this study, we first measured all 6 glypicans in plasma of patients with sepsis. Our finding that three of the glypicans, GPC 1, 3, and 4, are elevated in the plasma of sepsis patients indicates that these components of the glycocalyx may be involved in the pathophysiology of sepsis. Additionally, we found that GPC 1, 3, and 4 were elevated in patients who later developed organ failure, indicating that their increase in plasma is not a consequence of organ failure, but rather a preceding event.

A likely source of elevated GPC 1, 3, and 4 levels in sepsis is from the endothelial cell surface. Although all glypican expression decreases significantly in adulthood [31], GPC 1, 3, and 4 are still expressed in several organs and tissues in adults [26] and have been detected on the endothelium [32, 33]. The glycocalyx is shed extensively from the endothelium in sepsis and several of its components are found in the plasma during inflammatory conditions [10]. Alternately, mature monocyte-derived dendritic cells express GPC 3 while monocytes express GPC 4 [34]. Whether glypicans are shed from these cells in inflammatory conditions is unknown, but it is possible this could contribute to elevated GPC 3 and 4 in plasma. Glycocalyx components can also be shed from other cell types, including epithelial cells and fibroblasts, in response to inflammation and cell stress [35]. Since GPC 1, 3, and 4 expression is found in many organs [26], it is also possible that some of the elevated GPC 1, 3, and 4 is derived from other cell types and has diffused into the blood.

Shedding of glycocalyx components can occur in various ways [4]. Syndecans, as transmembrane proteins, can only be released by proteolytic cleavage. Heparan and chondroitin sulfate can be shed along with a proteoglycan core protein or can be removed by heparanases and chondroitinases. Mechanisms of glypican shedding are less well studied. In theory, glypicans can be released by three different mechanisms of shedding. First, glypicans are anchored to the cell membrane via a glycosylphosphatidylinositol (GPI) anchor, and so they can be shed from the membrane by phospholipases that cut the GPI moiety, freeing the whole protein core and the heparan sulfate chains from the membrane [36]. Phospholipase activity is altered in the plasma in sepsis [37, 38] and several bacteria can secrete phospholipases that could potentially sever the GPI anchor [39] so this shedding mechanism could be relevant in sepsis.

Glypicans can also be cleaved proteolytically by furin-like convertases [40]. Furin levels are unchanged in sepsis [41] so this mechanism of shedding is less likely. However, endoproteolytic cleavage of glypicans also occurs intracellularly prior to glypican trafficking to the cell surface [42]. This results in an N-terminal domain that is linked to the membrane-associated C-terminal domain only by disulfide bridges [40, 42]. Disulfide bonds are highly susceptible to breakage by reactive oxygen species (ROS) during changes in oxidative conditions [43], which could lead to the release of glypican N-terminal domains from the cell surface, leaving the C-terminal domain, which contains the heparan sulfate chains [40], still attached to the membrane. Sepsis is associated with massive increases in ROS and changes in the redox state of the blood and cells [44], and so this shedding mechanism could also be relevant in sepsis. Measurement of plasma glypicans can therefore potentially provide information about phospholipase- and ROS-mediated glycocalyx damage, which is not available from measurement of syndecan, hyaluronan or heparan sulfate levels.

The strengths of this study lie in our measurement of different glypican species to determine the relevant players in sepsis. We used commercial ELISA kits and measurement was done while blinded to the clinical outcomes of the patients. Additionally the cohort was fairly large and the patients had a wide range of clinical diagnoses and disease severities. A limitation of this study is that we were not able to distinguish between glypicans produced by the endothelium or by other cells. Additionally we did not examine whether increased plasma glypican levels are due to glycocalyx shedding, which may compromise endothelial barrier function, or whether they simply reflect increased turnover of the glycocalyx with no effect on barrier function. Recent experimental data from mice suggests that sepsis both induces shedding and impairs regeneration of the glycocalyx [45]. Lastly, the glypican ELISAs used polyclonal antibodies to the whole glypican protein, making it impossible to distinguish between glypicans shed by cleavage of the GPI anchor or the disulfide bridges. The development of ELISAs specific to the different cleavage products would be required to determine which form of each glypican is present in plasma and to indicate which shedding mechanisms are relevant during sepsis.

## Conclusions

We report for the first time that glypican 1, 3, and 4 levels are elevated in the plasma of patients with sepsis compared to those with infection without organ failure. GPC 1, 3, and 4 levels are associated with markers of inflammation and disease severity, and with plasma levels of other glycocalyx-related proteins. A greater understanding of glypican shedding in sepsis could provide insights into the mechanisms that lead to sepsis-associated glycocalyx damage.

## Additional file

Additional file 1: **Figure S1.** Pilot experiment measuring Glypican levels in sepsis patients; Description of data: GPC 1, 3 and 4 levels in the pilot cohort of patients with sepsis compared to healthy controls. (DOCX 512 kb)

## Abbreviations

ApoM: Apolipoprotein M
ARDS: Acute respiratory distress syndrome
CRP: C-reactive protein
ELISA: Enzyme-linked immunosorbent assay
GPC: Glypican
GPI: Glycosylphosphatidylinositol
HBP: Heparin binding protein
IL-6: Interleukin 6
PCT: Procalcitonin
ROC: Receiver operating characteristic
ROS: Reactive oxygen species
S1P: Sphingosine-1-phosphate
SDC: Syndecan
SIRS: Systemic inflammatory response syndrome

## References

[1] Singer M, Deutschman CS, Seymour CW (2016). The third international consensus definitions for sepsis and septic shock (Sepsis-3). J Am Med Assoc.

[2] Opal SM, van der Poll T (2015). Endothelial barrier dysfunction in septic shock. J Intern Med.

[3] Ince C, Mayeux PR, Nguyen T (2016). The endothelium in sepsis. SHOCK.

[4] Reitsma S, Slaaf DW, Vink H (2007). The endothelial glycocalyx: composition, functions, and visualization. Pflugers Arch.

[5] Chelazzi C, Villa G, Mancinelli P (2015). Glycocalyx and sepsis-induced alterations in vascular permeability. Crit Care.

[6] Alphonsus CS, Rodseth RN (2014). The endothelial glycocalyx: a review of the vascular barrier. Anaesthesia.

[7] Bansch P, Nelson A, Ohlsson T, Bentzer P (2011). Effect of charge on microvascular permeability in early experimental sepsis in the rat. Microvasc Res.

[8] Reitsma S, Slaaf DW, Vink H et al The endothelial glycocalyx: composition, functions, and visualization. 10.1007/s00424-007-0212-8 10.1007/s00424-007-0212-8 PMC1915585 17256154

[9] Iozzo RV, Schaefer L (2015). Proteoglycan form and function: a comprehensive nomenclature of proteoglycans. J Int Soc Matrix Biol.

[10] Schött U, Solomon C, Fries D, Bentzer P (2016). The endothelial glycocalyx and its disruption, protection and regeneration: a narrative review. Scand J Trauma Resusc Emerg Med.

[11] Park PW, Foster TJ, Nishi E (2004). Activation of syndecan-1 ectodomain shedding by Staphylococcus aureus α-toxin and β-toxin. J Biol Chem.

[12] Ibberson CB, Jones CL, Singh S (2014). Staphylococcus aureus hyaluronidase is a CodY-regulated virulence factor. Infect Immun.

[13] Chen Y, Hayashida A, Bennett AE (2007). Streptococcus pneumoniae sheds syndecan-1 ectodomains through ZmpC, a metalloproteinase virulence factor. J Biol Chem.

[14] Nelson A, Johansson J, Tydén J, Bodelsson M (2017). Circulating syndecans during critical illness. APMIS.

[15] Anand D, Ray S, Srivastava LM, Bhargava S (2016). Evolution of serum hyaluronan and syndecan levels in prognosis of sepsis patients. Clin Biochem.

[16] Johansson PI, Stensballe J, Rasmussen LS, Ostrowski SR (2011). A high admission syndecan-1 level, a marker of endothelial glycocalyx degradation, is associated with inflammation, protein C depletion, fibrinolysis, and increased mortality in trauma patients. Ann Surg.

[17] Rehm M, Zahler S, Lötsch M (2004). Endothelial glycocalyx as an additional barrier determining extravasation of 6% hydroxyethyl starch or 5% albumin solutions in the coronary vascular bed. Anesthesiology.

[18] Chappell D, Jacob M, Hofmann-Kiefer K (2007). Hydrocortisone preserves the vascular barrier by protecting the endothelial glycocalyx. Anesthesiology.

[19] Chen C, Huang X, Ying Z (2017). Can glypican-3 be a disease-specific biomarker?. Clin Transl Med.

[20] Linder A, Fisher J (2015) Plasma glypican-4 levels are associated with disease severity in ED patients with severe Sepsis and septic shock. Open Forum Infect Dis 2. 10.1093/ofid/ofv133.120

[21] Bone RC, Alan Fein CM, Robert Balk FA (1992). Definitions for sepsis and organ failure and guidelines for the use of innovative therapies in sepsis. The ACCP/SCCM Consensus Conference Committee. American College of Chest Physicians/Society of Critical Care Medicine. Chest.

[22] Linder A, Christensson B, Herwald H (2009). Heparin-binding protein: an early marker of circulatory failure in sepsis. Clin Infect Dis.

[23] Frej C, Linder A, Happonen KE (2016). Sphingosine 1-phosphate and its carrier apolipoprotein M in human sepsis and in *Escherichia coli* sepsis in baboons. J Cell Mol Med.

[24] Kumaraswamy SB, Linder A, Akesson P, Dahlback B (2012). Decreased plasma concentrations of apolipoprotein M in Sepsis and systemic inflammatory response syndromes. Crit Care.

[25] Tapper H, Karlsson A, Mörgelin M (2002). Secretion of heparin-binding protein from human neutrophils is determined by its localization in azurophilic granules and secretory vesicles. Blood.

[26] Filmus J (2001). Glypicans in growth control and cancer. Glycobiology.

[27] Sonneville R, Verdonk F, Rauturier C (2013). Understanding brain dysfunction in sepsis. Ann Intensive Care.

[28] dos Santos CC, Gattas DJ, Tsoporis JN (2010). Sepsis-induced myocardial depression is associated with transcriptional changes in energy metabolism and contractile related genes: a physiological and gene expression-based approach. Crit Care Med.

[29] Fisher J, Russell JA, Bentzer P (2017). Heparin-binding protein (HBP): a causative marker and potential target for heparin treatment of human sepsis-induced acute kidney injury. SHOCK.

[30] Zeng Y, Adamson RH, Curry F-RE, Tarbell JM (2014). Sphingosine-1-phosphate protects endothelial glycocalyx by inhibiting syndecan-1 shedding. AJP Hear Circ Physiol.

[31] Song HH, Filmus J (2002). The role of glypicans in mammalian development. Biochim Biophys Acta.

[32] Patterson AM, Cartwright A, David G (2008). Differential expression of syndecans and glypicans in chronically inflamed synovium. Ann Rheum Dis.

[33] Lundqvist K, Schmidtchen A (2001). Immunohistochemical studies on proteoglycan expression in normal skin and chronic ulcers. Br J Dermatol.

[34] Wegrowski Y, Milard A-L, Kotlarz G (2006). Cell surface proteoglycan expression during maturation of human monocytes-derived dendritic cells and macrophages. Clin Exp Immunol.

[35] Manon-Jensen T, Itoh Y, Couchman JR (2010). Proteoglycans in health and disease: the multiple roles of syndecan shedding. FEBS J.

[36] Traister A, Shi W, Filmus J (2008). Mammalian Notum induces the release of glypicans and other GPI-anchored proteins from the cell surface. Biochem J.

[37] Green J-A, Smith GM, Buchta R (1991). Circulating phospholipase A2 activity associated with sepsis and septic shock is indistinguishable from that associated with rheumatoid arthritis. Inflammation.

[38] Rhode H, Lopatta E, Schulze M (1999). Glycosylphosphatidylinositol-specific phospholipase D in blood serum: is the liver the only source of the enzyme?. Clin Chim Acta.

[39] Schmiel DH, Miller VL (1999). Bacterial phospholipases and pathogenesis. Microbes Infect.

[40] Filmus J, Capurro M, Rast J (2008). Glypicans. Genome Biol.

[41] Ranta N, Turpeinen H, Oksanen A (2015). The plasma level of proprotein convertase FURIN in patients with suspected infection in the emergency room: a prospective cohort study. Scand J Immunol.

[42] De Cat B, Muyldermans S-Y, Coomans C (2003). Processing by proprotein convertases is required for glypican-3 modulation of cell survival, Wnt signaling, and gastrulation movements. J Cell Biol.

[43] Cremers CM, Jakob U (2013). Oxidant sensing by reversible disulfide bond formation. J Biol Chem.

[44] Huet O, Dupic L, Harrois A, Duranteau J (2011). Oxidative stress and endothelial dysfunction during sepsis. Front Biosci (Landmark Ed).

[45] Yang Y, Haeger SM, Suflita MA (2017). Fibroblast growth factor signaling mediates pulmonary endothelial Glycocalyx reconstitution. Am J Respir Cell Mol Biol.

