# Supplementary material for: Elevated plasma glypicans are associated with organ failure in patients with infection
Source: Intensive Care Med Exp. 2019 Jan 7;7:2. doi: 10.1186/s40635-018-0216-z (PMC6323058; doi:10.1186/s40635-018-0216-z)
Supplement: Supplementary file 1 — Figure S1. Pilot experiment measuring Glypican levels in sepsis patients; Description of data: GPC 1, 3 and 4 levels in the pilot cohort of patients with sepsis compared to healthy controls. (DOCX 512 kb) [file 40635_2018_216_MOESM1_ESM.docx]

**
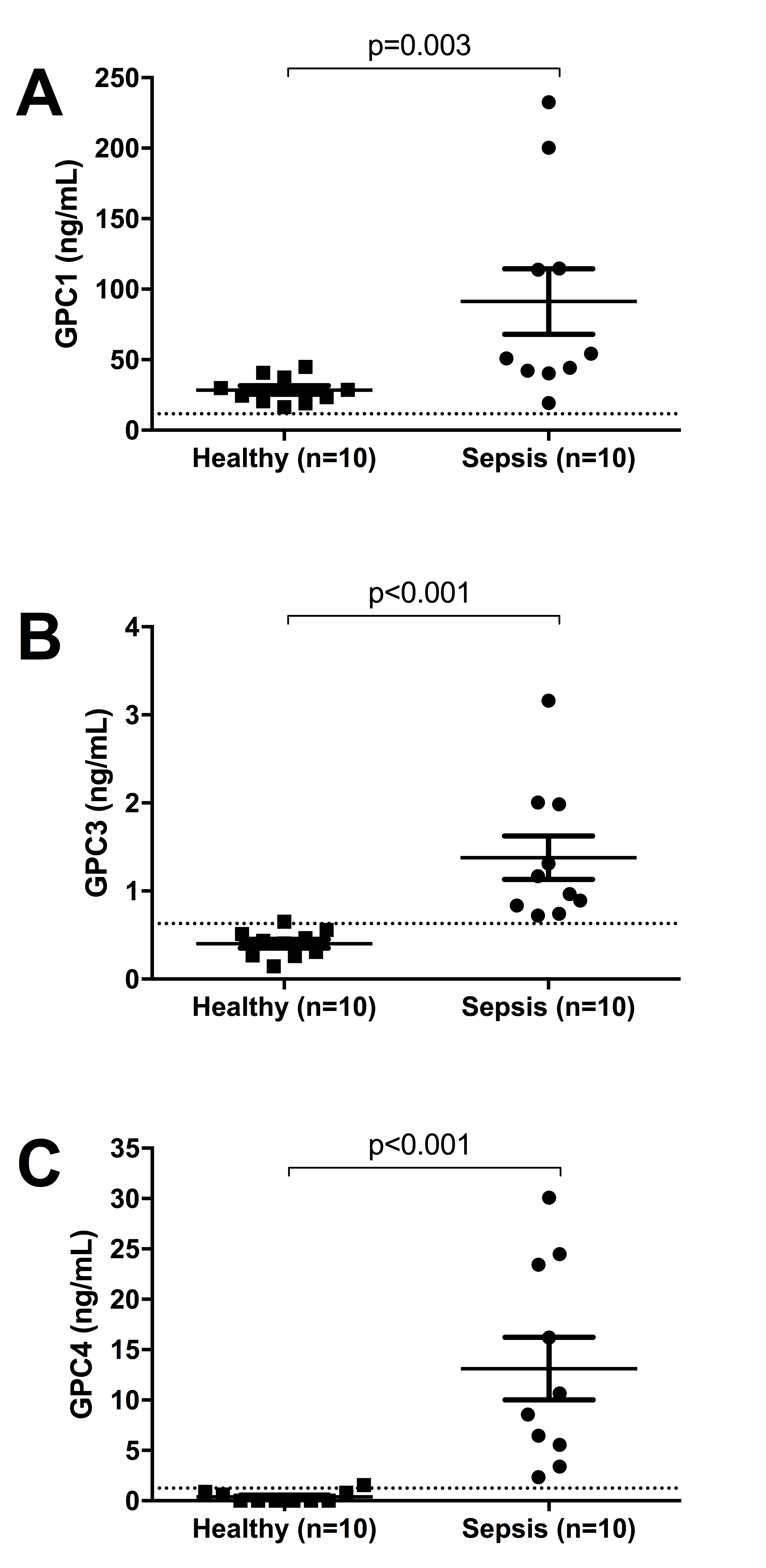
**

**Additional file 1: Figure S1. Pilot experiment measuring Glypican levels in sepsis patients.** GPC1, 3 and 4 levels were measured by ELISA in patients with sepsis and healthy controls. Differences between these groups were determined by Mann-Whitney test. The lower limit of detection of each assay is shown as a dashed line.
